# Three-dimensional trunk adjustment based on surface markers in treating mild to moderate adolescent idiopathic scoliosis

**DOI:** 10.3389/fped.2026.1683567

**Published:** 2026-02-05

**Authors:** Yanli Yao, Guangyan Han, Haibin Guo, Chao Ye, Xixi Wang, Hui Wang

**Affiliations:** 1 Department of Rehabilitation Medicine, Affiliated Changzhou Children’s Hospital of Nantong University, Changzhou, Jiangsu, China; 2 Department of Orthopedics, Affiliated Changzhou Children’s Hospital of Nantong University, Changzhou, Jiangsu, China

**Keywords:** 3D adjustment, adolescent idiopathic scoliosis, angle of trunk rotation, Cobb angle, surface markers

## Abstract

**Background:**

Adolescent idiopathic scoliosis (AIS) has both physical and psychological impacts, requiring multidisciplinary non-surgical interventions. Incorporating three-dimensional (3D) adjustments may enhance the efficacy of traditional exercises. This study aimed to explore the clinical effect of integrating trunk 3D adjustments with conventional rehabilitation exercises in the treatment of children with mild to moderate AIS.

**Methods:**

This study enrolled AIS patients treated at our hospital. Participants were assigned to either the combined therapy group, receiving trunk 3D adjustment and traditional rehabilitation, or the traditional therapy group, receiving traditional rehabilitation alone. The primary outcomes were the Cobb angle and angle of trunk rotation (ATR).

**Results:**

A total of 46 children were included, 23 in each group, with median ages of 12 and 13 years for the traditional and combined therapy groups, respectively. Baseline characteristics showed no differences (all *P* > 0.05). After 6 months, the combined therapy group significantly improved in Cobb angle (7.00 vs. 10.00 degrees, *P* = 0.024) and ATR (3.26 ± 1.98 vs. 4.39 ± 1.78, *P* = 0.048), and showed better outcomes in forward head posture, shoulder asymmetry, and pelvic tilt (all *P* < 0.05).

**Conclusion:**

The integration of trunk 3D adjustment with conventional rehabilitation training effectively improves Cobb angle and ATR in mild to moderate AIS patients, highlighting the importance and potential of body alignment awareness for clinical treatment.

## Introduction

Adolescent idiopathic scoliosis (AIS) is a musculoskeletal disorder of unknown etiology (1). This condition not only involves a spinal deformity but also commonly presents with asymmetrical shoulder heights, razorback deformity, thoracic distortion, and pelvic tilt, all contributing to a three-dimensional (3D) imbalance. Without timely treatment, AIS can lead to complications such as pain, pulmonary dysfunction, and in severe cases, irreversible damage to respiratory function and the spinal cord (2). In addition, significant psychological distress, including anxiety, feelings of inferiority, and social behavioral issues, may occur (3).

According to the Society on Scoliosis Orthopedic and Rehabilitation Treatment (SOSORT) guidelines, the primary goal in AIS management is to enhance aesthetic outcomes (4). Non-surgical treatment, recommended for patients with a Cobb angle of less than 45°, aims to delay curvature progression and reduce the necessity for surgical intervention (5). Such treatments include exercise therapy, bracing, and manual therapy. Currently, AIS exercise regimens are collaboratively formulated by a team—including orthopedic surgeons, orthoptists, nurses, rehabilitation therapists, and parents—tailored to the needs of each patient.

Among non-surgical interventions, internationally recognized physiotherapeutic scoliosis-specific exercises (PSSE) methods, such as the Schroth and SEAS approaches, have established evidence for 3D spinal correction and are endorsed by SOSORT and the Scoliosis Research Society (SRS) (6–8). These approaches are designed to actively correct spinal deformities by combining targeted breathing techniques, rotational angular breathing, and precise postural exercises across the coronal, sagittal, and transverse planes (9, 10). They typically require intensive, individualized supervision by highly trained therapists to ensure correct execution and optimize spinal realignments. The experience of the therapist, the characteristics of the child, and support from the parents are major influences in the success of the Schroth program (11, 12). However, despite this guidance, patients—particularly adolescents—may reduce compliance or adherence due to limited awareness of the dynamic relationship between trunk orientation and spinal alignment (13, 14). This reliance on therapist-directed cues can reduce the degree of autonomous postural control and limit the generalizability of PSSE outside clinical settings.

Conventional exercise therapies often overlook the patient's personal perception and lack standardized trunk correction metrics in 3D space (15). To address this gap, a novel approach—referred to as “3D Trunk Adjustment”—has been developed to enhance patient engagement in postural control. This method cultivates “framework awareness” in children, emphasizing that the trunk and spine are distinct but interdependent structures. Understanding this relationship allows patients to perceive how trunk movements can influence spinal alignment and how spinal adjustments can affect overall trunk posture. By internalizing this framework, children gain an intuitive sense of their own body alignment, which can guide self-directed corrections in three-dimensional space. Such an approach provides a foundation for patients to manage posture independently and may help limit the progression of spinal curvature while complementing conventional rehabilitation exercises. Our clinical observations indicate that trunk 3D adjustments, referenced by surface markers, significantly bolsters the patient's awareness of body alignment. This method utilizes body surface markers for correctional guidance and visual feedback techniques such as photography and mirror observation to improve spatial body awareness, enhance control over body movements, and ameliorate trunk deformities. Therefore, this study investigates the clinical effect of integrating trunk 3D adjustments with traditional rehabilitation exercises to ameliorate postural abnormalities in AIS.

## Materials and Methods

### Study design and participants

This quasi-experimental study was conducted on patients with AIS treated in the Rehabilitation Department of our hospital between July 2020 and June 2023. AIS diagnosis met the standards of the SRS (16), based on standing full-spine coronal X-ray demonstrating spinal lateral curvature with a Cobb angle greater than 10° and axial rotation. The inclusion criteria were as follows: (1) established diagnostic guidelines for AIS must be met by patients; (2) age between 9 and 18 years; (3) Cobb angle ranging from 10° to 25°; (4) normal visual function; (5) no history of surgical interventions or brace treatments for scoliosis; and (6) voluntary informed consent signed by both the patients and guardians. The exclusion criteria were as follows: (1) diagnosed mental illness; (2) severe cardiopulmonary dysfunction; (3) secondary scoliosis arising from congenital abnormalities in nerves, muscles, or vertebrae; and (4) leg length discrepancy greater than 1 cm. Discontinuation criteria stated that patients may be withdrawn from the study if they were unable to actively cooperate with the treatment regimen, if they received other treatments that could interfere with the study's efficacy without prior authorization, or if they choose to abandon the treatment protocol. This study was approved by the Ethics Committee of Changzhou Children's Hospital (No. 2020Y016). Written informed consent was obtained from all participants.

### Conventional rehabilitation

Both the traditional therapy group and the combined therapy group completed conventional rehabilitation training, conducted 3–5 times per week over a 6-month period (17). This training regimen comprised scapular control exercises and core training. Scapular control exercises included Y Movement, T Movement, W Movement, and L Movement, designed to strengthen and stabilize the scapulae (details provided in Supplementary material).

### Intervention

Prior to intervention, the diagnosis and follow-up evaluation of adolescent idiopathic scoliosis were based on standing full-spine coronal radiographs. The Cobb angle was measured using standard techniques by experienced rehabilitation therapists. The combined therapy group underwent a combination of conventional rehabilitation training and targeted trunk 3D adjustments, based on body surface markers. Prior to beginning the rehabilitation process, a chief rehabilitation therapist provided specialized one-on-one offline training for both parents and children in the combined therapy group. This training focused on teaching the children how to identify parts of their trunk using surface markers and how to rectify poor posture from a 3D perspective (18). Following training, detailed one-on-one assessments were conducted in which children actively described points, lines, and surfaces, and demonstrated their understanding of posture adjustment. Parents participated in three such training and assessment sessions before commencing the formal intervention.

This regimen was performed twice daily for a duration of 6 months. The training emphasized the correct alignment of various anatomical points and lines, which were identified as follows (Supplementary Figures 1, 2):

a) Points: Soles, second toes, midpoints of ankles, patellae, pelvis, navel, sternum, chin, nose, ears, acromion, greater trochanter, knees, and lateral ankles.
b) Lines: anterior midline: A vertical line running from the nose, chin, chest, navel, pubic symphysis, between the knees, and between the ankles. Anterior axillary line: A vertical line from the front edge of the armpit to the side of the pelvis. Rib line: A horizontal line at the lowest point of the 10th rib. Anterior superior iliac spine line: A horizontal line at the level of the anterior superior iliac spines.
c) Surfaces: Adjustments were made based on the coronal, horizontal, and sagittal planes to correct imbalances. Coronal plane: Focused on lateral shifts, aligning the anterior midline and both anterior axillary lines with the pelvis lateral lines. Horizontal plane: Corrected rotations, aligning the toes, patellae, iliac spines, rib lines, nipples, and shoulders. Sagittal plane: Targeted changes in physiological curves, aligning the pelvis in a neutral position and ensuring a straight line from the ears, acromion, greater trochanter, and lateral ankle.

**Figure 1:**
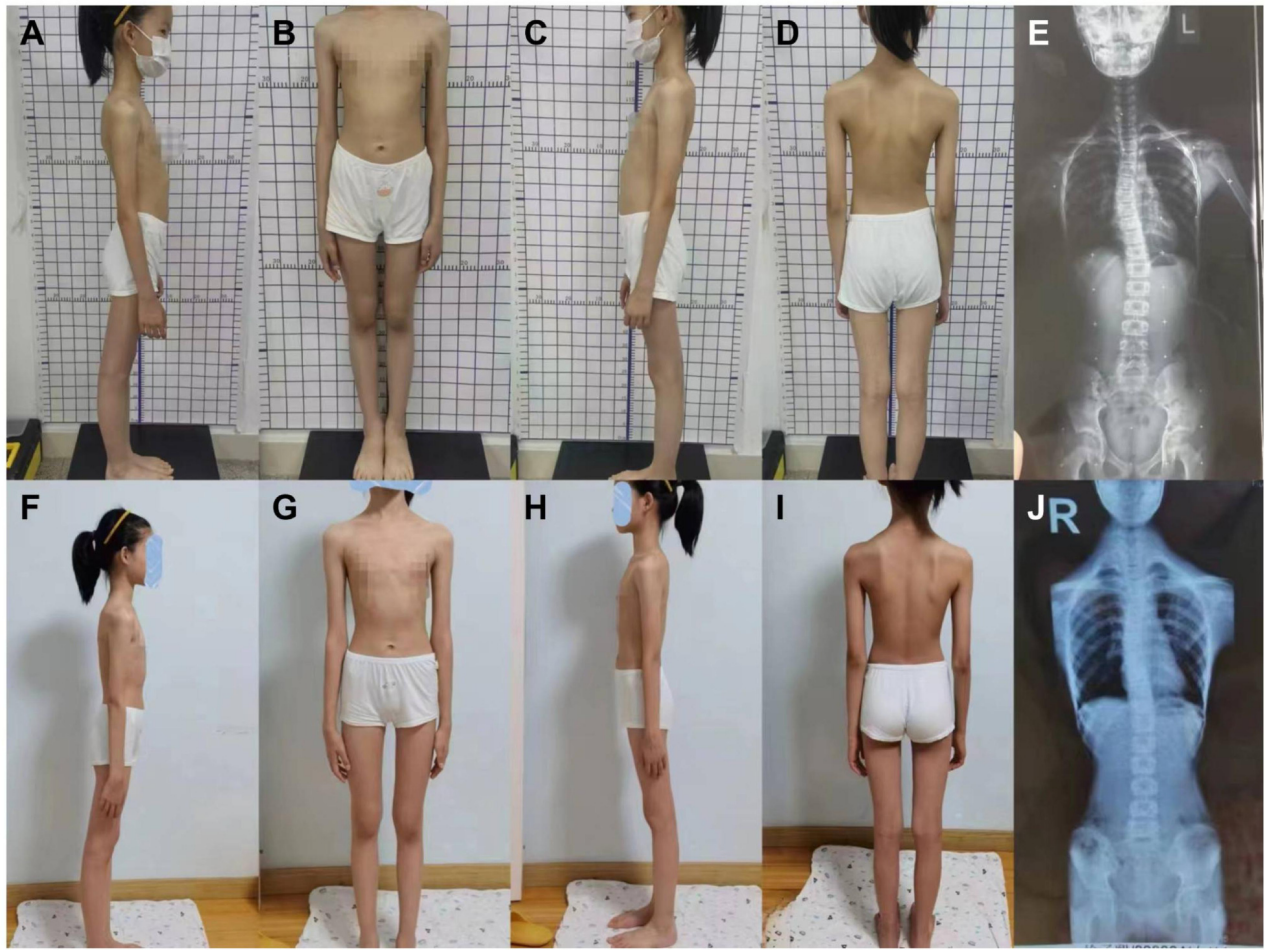
Images of a 10-year-old girl from the combined therapy group before treatment **(A–E)** and after treatment **(F–J)**. The patient was diagnosed with scoliosis, with a Cobb angle of 15° and an ATR of 8°. The pretreatment images **(A–E)** were taken in September 2020, including **(A)** right-side standing posture view, **(B)** frontal view, **(C)** left-side standing posture view, **(D)** back view, and **(E)** full-length coronal X-ray of the spine. The posttreatment images **(F–J)** were taken in March 2021, showing **(F)** right-side standing posture view, **(G)** frontal view, **(H)** left-side standing posture view, **(I)** back view, and **(J)** full-length coronal X-ray of the spine.

**Figure 2:**
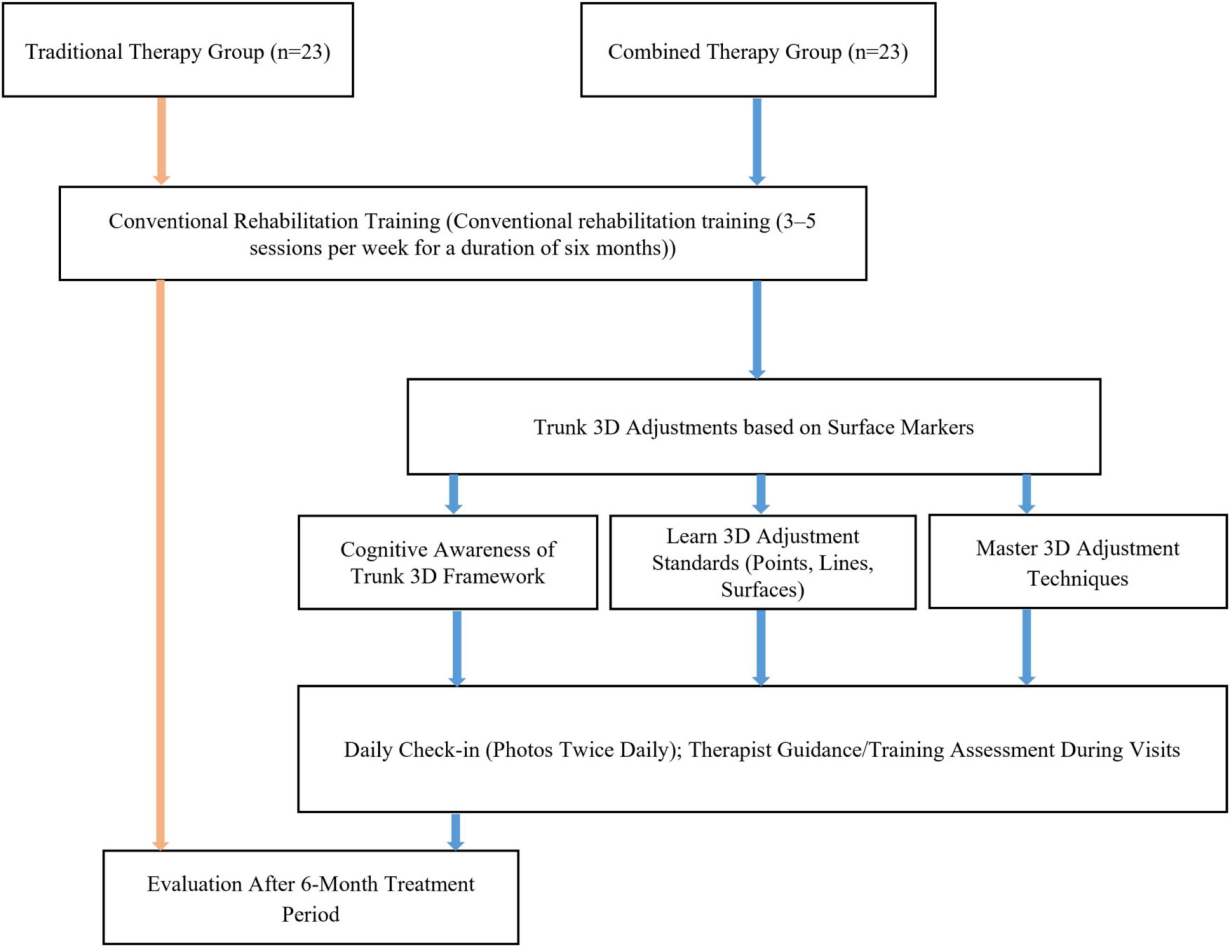
Overall study workflow in the traditional therapy and the combined therapy groups.

Each fixed posture was photographed and maintained for 5–15 s, with daily front, back, and side photos taken twice (Figure 1). Parents took the first set of photos and applied a grid overlay to clearly visualize 3D trunk changes in the coronal plane (shoulder height, ribcage offset, and pelvic height), horizontal plane (waistline, arm position), and sagittal plane (pelvic tilt). Children and parent then observed the photos, identified issues, and performed qualitative self-adjustments across the coronal, sagittal, and horizontal planes and made 3D posture adjustments. A second photo was then taken and sent to the therapist for remote guidance. In addition, children visited the hospital every 1–2 weeks for direct evaluation and adjustment guidance. This combination of home-based visual feedback and periodic in-person supervision enabled children to gradually develop a “posture awareness framework,” fostering self-correction and limiting scoliosis progression. In subsequent treatments, the therapist would guide children on correcting coronal, sagittal, and horizontal plane issues. Basic posture practice was conducted daily for 6 months. The overall study workflow is illustrated in Figure 2.

### Data collection and outcome

Evaluations were conducted for both groups prior to treatment and after a six-month treatment period. Measurements were meticulously taken by two senior rehabilitation therapists, each with over three years of experience in scoliosis management—one responsible for performing the measurements and the other responsible for recording and photographing, including coronal X-rays for Cobb angle measurements and posture assessment photographs.

The primary outcomes were the Cobb angle and the angle of trunk rotation (ATR). The Cobb angle was determined by drawing extension lines from the upper endplate of the highest vertebra and the lower endplate of the lowest vertebra involved in the scoliosis curve (19). The angle formed by these lines, or their perpendiculars, is considered the gold standard for diagnosing scoliosis, with angles greater than 10° indicating the presence of the condition (4). ATR was measured during the Adam's forward bend test (20). Patient stood with feet pelvis-width apart and hands together, slowly bending forward to a 90° angle. A scoliometer was placed on the most deformed area without applying pressure to accurately record the rotational angle (21). Inter-rater reliability for Cobb angle measurements was assessed between the two experienced therapists, yielding excellent agreement (ICC = 0.907, 95% CI 0.839–0.947, *P* < 0.001).

The secondary outcome included basic posture indicators, such as forward neck tilt, shoulder height asymmetry, and pelvic tilt. Basic posture indicators were assessed using a posture diagram and classified into mild, moderate, or severe categories based on the severity of the posture, with scoring as follows: no posture problem (0 points), mild (1 point), moderate (3 points), and severe (5 points), with a total possible score ranging from 0 to 15 points. Forward neck tilt was assessed from the side view by checking the vertical alignment of the ears, acromion, greater trochanter, lateral knee line, and lateral ankle with the central axis of the posture diagram (22). Deviations were scored based on the distance from the acromion, with 0–3 cm considered mild, >3–5 cm moderate, and >5 cm severe. Shoulder height asymmetry was evaluated from both front and back views to determine if the shoulders were level. Asymmetry was scored based on the differences in height on the same side across both views: 0–2 cm considered mild, >2–4 cm moderate, and >4 cm severe (23). Pelvic tilt was observed from the side view to determine the forward rotation of the pelvis, with scoring similar to other posture assessments (24).

### Statistical analysis

All statistical analyses were performed using SPSS 26.0 (IBM, Armonk, NY, USA). Categorical variables were expressed as frequencies (percentages) and analyzed using Pearson's chi-squared test or Fisher's exact test. Continuous variables adhering to a normal distribution were presented as mean and standard deviation (SD). Between-group comparisons were conducted using the independent samples *t*-test, while changes before and after the intervention were assessed using the paired *t*-test. For continuous variables that follow a skewed distribution, values were expressed as medians along with the 25th and 75th percentiles. Between-group comparisons were performed for these variables using the Mann–Whitney *U*-test, while within-group changes over time were evaluated using the Wilcoxon signed-rank test. Statistical significance was established as a two-sided *P* value of less than 0.05.

## Results

A total of 50 AIS patients meeting the inclusion criteria and providing informed consent were enrolled and divided into the traditional therapy group and the combined therapy group. The traditional therapy group included 24 patients, while the combined therapy group included 26 patients. Due to midtreatment adoption of other therapies (e.g., osteopathy, acupuncture), one patient was lost from the traditional therapy group and three patients from the combined therapy group, resulting in a total of 46 patients completing the study, with 23 patients in each group.

The median age was 12 years (10.50–12.50) in the traditional therapy group and 13 years (12.00–14.00) in the combined therapy group, with no significant difference (*P* = 0.074). Females made up 87.0% of the traditional therapy group and 91.3% of the combined therapy group, showing comparable gender representation (*P* > 0.999). The average heights (153.78 ± 8.73 cm vs. 157.35 ± 8.13 cm, *P* = 0.159), average weights (39.67 ± 9.85 kg vs. 45.33 ± 10.47 kg, *P* = 0.066), and distributions of scoliosis types (*P* > 0.999) showed no statistical differences between the two groups. At baseline, Cobb angles (*P* = 0.293), ATR medians (*P* = 0.178), forward head posture (*P* = 0.239), shoulder asymmetry (*P* = 0.161), and pelvic tilt (*P* = 0.749) showed no significant differences between the traditional therapy and combined therapy groups (Table 1).

**Table 1:** Baseline characteristics.

Characteristics	Traditional therapy group (*n* = 23)	Combined therapy group (*n* = 23)	*P*
Age (years)	12.00 [10.50, 12.50]	13.00 [12.00, 14.00]	0.074
Gender, *n* (%)			
Female	20 (87.0)	21 (91.3)	>0.999
Male	3 (13.0)	2 (8.7)	
Height (cm)	153.78 (8.73)	157.35 (8.13)	0.159
Weight (kg)	39.67 (9.85)	45.33 (10.47)	0.066
Type of scoliosis, *n* (%)			
Thoracic curve	4 (17.4)	4 (17.4)	>0.999
Thoracolumbar curve	12 (52.2)	12 (52.2)	
Right thoracic-left lumbar curve	5 (21.7)	5 (21.7)	
Lumbar curve	2 (8.7)	2 (8.7)	
Cobb angle (°)	13.92 (3.09)	15.13 (4.49)	0.293
ATR angle (°)	6.00 [6.00, 9.00]	9.00 [6.50, 10.00]	0.178
Forward head posture	1.00 [0.00, 1.00]	1.00 [1.00, 1.00]	0.239
Shoulder asymmetry	1.00 [1.00, 1.00]	1.00 [1.00, 1.00]	0.161
Pelvic tilt	1.00 [1.00, 3.00]	1.00 [1.00, 3.00]	0.794

After 6 months of treatment, the Cobb angle demonstrated improvement, with the combined therapy group showing a median of 7.00 degrees compared to 10.00 degrees in the traditional therapy group (*P* = 0.024). Similarly, the ATR angle was significantly lower in the combined therapy group (3.26 ± 1.98°) compared to the traditional therapy group (4.39 ± 1.78°), with a *P* value of 0.048. Forward head posture (*P* = 0.002), shoulder asymmetry (*P* = 0.04), and pelvic tilt (*P* = 0.019) all also showed significant improvement in the combined therapy group compared to the traditional therapy group (Table 2).

**Table 2:** Clinical outcomes after treatments.

Outcomes	Traditional therapy group (*n* = 23)	Combined therapy group (*n* = 23)	*P*
Cobb angle (°)	10.00 [8.00, 12.50]	7.00 [4.50, 11.00]	0.024
ATR angle (°)	4.39 (1.78)	3.26 (1.98)	0.048
Forward head posture	0.00 [0.00, 1.00]	0.00 [0.00, 0.00]	0.002
Shoulder asymmetry	1.00 [0.00, 1.00]	0.00 [0.00, 1.00]	0.04
Pelvic tilt	0.00 [0.00, 0.00]	0.00 [0.00, 0.00]	0.019

To improve transparency and demonstrate the magnitude of treatment effects, pre- and postintervention outcomes for both groups, along with within-group comparisons, are presented in Supplementary Table 1. As shown, both groups exhibited statistically significant improvements in Cobb angle and ATR after 6 months of intervention (all within-group *P* < 0.001). The combined therapy group demonstrated a greater absolute reduction in Cobb angle and ATR compared with the traditional therapy group. These data provide a clearer depiction of baseline values, posttreatment outcomes, and the extent of change over time, facilitating interpretation of both statistical and potential clinical significance.

## Discussion

The results of this study showed that integrating trunk 3D adjustments with traditional rehabilitation exercises led to significant improvements in Cobb angle, ATR angle, forward head posture, shoulder asymmetry, and pelvic tilt in AIS patients compared to traditional rehabilitation alone. These findings highlight the clinical efficacy of the combined therapeutic approach in treating AIS-related postural deformities.

This study demonstrated that employing trunk 3D adjustment therapy using surface markers as reference points markedly enhances posture correction in AIS patients by actively engaging the cognitive system. Previous research has shown increased brain activity in motor-related areas and decreased activity in lower limb muscles, leading to reduced cocontraction of calf muscles under stable posture conditions (25). Similarly, Beaudette et al. found that the sense of trunk position significantly influences posture control, while Celenay et al. indicated that enhancing trunk position sense aids in stabilizing posture (26, 27). This study supports the use of surface markers to help AIS patients become more aware of their bodies, perceive their center of gravity and trunk position, and adjust their posture within a 3D framework, thereby improving body alignment and trunk control. Notably, this approach led to a more significant improvement in the Cobb angle compared to traditional rehabilitation therapy, potentially due to the neuromuscular control mechanisms involved in spinal curvature management. Adolescents with AIS, being in a critical phase of growth, experience disruptions in the dynamic stability control system of the spine due to AIS, leading to altered posture control (28). Adjustments in posture can be achieved by modulating the input and central cognitive systems within the feedback pathway, which may also relate to biomechanical changes in the spine (29). Prolonged uneven loading on the vertebral bodies can exacerbate spinal curvature (30). This is particularly relevant for Chinese adolescents who face high academic pressures and often study in poor postures, such as leaning forward, which predisposes them to spinal deformities. Thus, daily management of trunk posture in 3D space is essential (31).

The theoretical mechanism underlying 3D trunk adjustment is grounded in sensorimotor integration, motor learning, and biomechanical load redistribution. Adolescents with idiopathic scoliosis commonly exhibit impaired trunk proprioception, altered postural control, and compensatory movement strategies, which may contribute to curve progression (32). By using surface markers and visual feedback, the 3D trunk adjustment approach enhances trunk position sense and spatial body awareness across the coronal, sagittal, and transverse planes, facilitating recalibration of internal postural representations through augmented feedback mechanisms (33). The concept of “framework awareness” further emphasizes hierarchical control, whereby a stable trunk framework is first established to guide more selective spinal movement along the midline, thereby reducing excessive trunk compensation and improving neuromuscular coordination. Repeated low-load, self-directed postural correction during daily activities may promote neuroplastic adaptation and reduce asymmetric mechanical loading on the growing spine, a known contributor to scoliosis progression (34). In this context, 3D trunk adjustment functions as a form of internalized or “cognitive orthosis,” extending posture regulation beyond supervised therapy sessions. It complements established PSSE approaches by enhancing patient autonomy and facilitating the transfer of corrective strategies into real-world settings (35, 36).

When compared with internationally recognized PSSE methods, such as Schroth and SEAS, the conceptual focus of the present intervention differs in several important ways. PSSE approaches are strongly supported by SOSORT and SRS guidelines and have demonstrated efficacy in reducing curve progression through therapist-guided 3D spinal correction, rotational breathing, and curve-specific exercises (36, 37). However, these methods typically require intensive therapist supervision and precise corrective cues. Adolescents may develop compensatory trunk movements if trunk-spine relationships are not sufficiently internalized (38). In contrast, the current approach places initial emphasis on cultivating “framework awareness,” enabling patients to recognize the trunk and spine as related but distinct structures. This theoretical distinction may reduce compensatory trunk strategies during active correction and support autonomous postural control outside the clinical setting. Similar concepts have been explored in body awareness-based therapies, which have shown promise in improving posture and functional outcomes in adolescents with AIS (39).

The primary strength and novelty of this study lie in its emphasis on body framework awareness as a prerequisite for spinal correction. Unlike conventional rehabilitation approaches that focus predominantly on exercise execution or therapist-driven correction, this intervention trains children to recognize that the trunk and spine are related but distinct structures: Trunk asymmetry can influence spinal deviation, and spinal deformity can, in turn, alter trunk alignment (40). By developing this framework awareness, children effectively form an internalized “awareness brace,” which helps limit further curve progression during daily activities outside supervised therapy sessions. More importantly, during scoliosis-specific adjustment training, children learn to focus on active spinal movement along the midline, consciously reducing trunk compensation and initiating correction from the spine rather than relying solely on external posture correction (41). Another important strength of this approach is its non-invasive and highly reproducible nature. The intervention relies on simple tools—surface markers, photographic feedback, and therapist-guided qualitative cues—without requiring complex equipment or advanced imaging. This makes the method feasible for long-term home-based application and broad clinical dissemination. Together, these features distinguish the present intervention from other rehabilitation-based approaches and highlight its potential value as a sustainable, patient-centered non-operative treatment for mild to moderate adolescent idiopathic scoliosis.

This study has several limitations. It utilized a quasi-experimental design rather than a randomized controlled trial (RCT), which may introduce selection bias. Furthermore, being a single-center study limits the generalizability of the findings. The relatively small sample size may affect the statistical power. The study focused on short-term outcomes over 6 months, leaving the long-term effects to be explored in future research. In addition, due to the nature of the intervention, blinding was difficult to achieve, potentially introducing observer bias. The posture scoring system used has not been formally validated, and its reliability and validity should be confirmed in future studies. An important limitation of this study is that skeletal maturity was not assessed using the Risser staging system. Risser stage is a well-recognized prognostic indicator for curve progression in adolescent idiopathic scoliosis and plays a critical role in interpreting treatment-related changes in Cobb angle (42). The absence of Risser staging limits the ability to fully distinguish the observed improvements from potential natural stabilization associated with growth deceleration. Although pre- and postintervention coronal radiographs demonstrated reductions in the distance between the C7 plumb line and the central sacral vertical line, as well as decreased displacement of the apical vertebra relative to the sacral midline—suggesting genuine postural and alignment changes beyond known Cobb angle measurement error—future studies should incorporate skeletal maturity assessment to better control for growth-related confounding and strengthen causal inference.

## Conclusion

Integrating trunk 3D adjustments with traditional rehabilitation exercises in adolescents with idiopathic scoliosis was associated with significant improvements in Cobb angle, ATR, forward head posture, shoulder asymmetry, and pelvic tilt compared to conventional rehabilitation alone. These findings suggest that incorporating cognitive trunk awareness and three-dimensional postural correction may enhance the effectiveness of non-operative AIS interventions. However, due to limitations such as the quasi-experimental design, small sample size, short-term follow-up, unblinded assessment, and the absence of Risser staging to account for skeletal maturity, the observed improvements should be interpreted with caution. While the intervention shows promise as a feasible, non-invasive, and home-applicable strategy for posture correction, further research using randomized controlled trials with larger cohorts, long-term follow-up, and incorporation of skeletal maturity measures is required to confirm its efficacy and generalizability.

## Data Availability

The original contributions presented in the study are included in the article/Supplementary Material, further inquiries can be directed to the corresponding author.

## Supplementary material

Supplementary Figure 1 Schematic illustration of surface marker placement and frame construction for three-dimensional trunk assessment. **(A)** Anterior view showing the placement of key surface markers. **(B)** Anterior view with markers connected to form the anterior reference frame, which allows clear visualization of coronal-plane deviations, including lateral translation of the pelvis, thorax, and head; within the frame concept, rotational abnormalities in the horizontal plane can also be inferred from the coronal-view image. **(C)** Lateral view showing the placement of key surface markers. **(D)** Lateral view with markers connected to form the sagittal reference frame, enabling assessment of sagittal-plane postural abnormalities, such as forward head posture, anterior pelvic tilt, and knee hyperextension. **(E)** Posterior view showing the placement of key surface markers. **(F)** Posterior view with markers connected to form the posterior reference frame, facilitating evaluation of posterior trunk alignment and asymmetry.

Supplementary Figure 2 Grid on photographs of coronal plane, horizontal plane and sagittal plane.
