# Supplementary figures and images for: Three-dimensional trunk adjustment based on surface markers in treating mild to moderate adolescent idiopathic scoliosis
Source: Front Pediatr. 2026 Feb 5;14:1683567. doi: 10.3389/fped.2026.1683567 (PMC12916605; doi:10.3389/fped.2026.1683567)

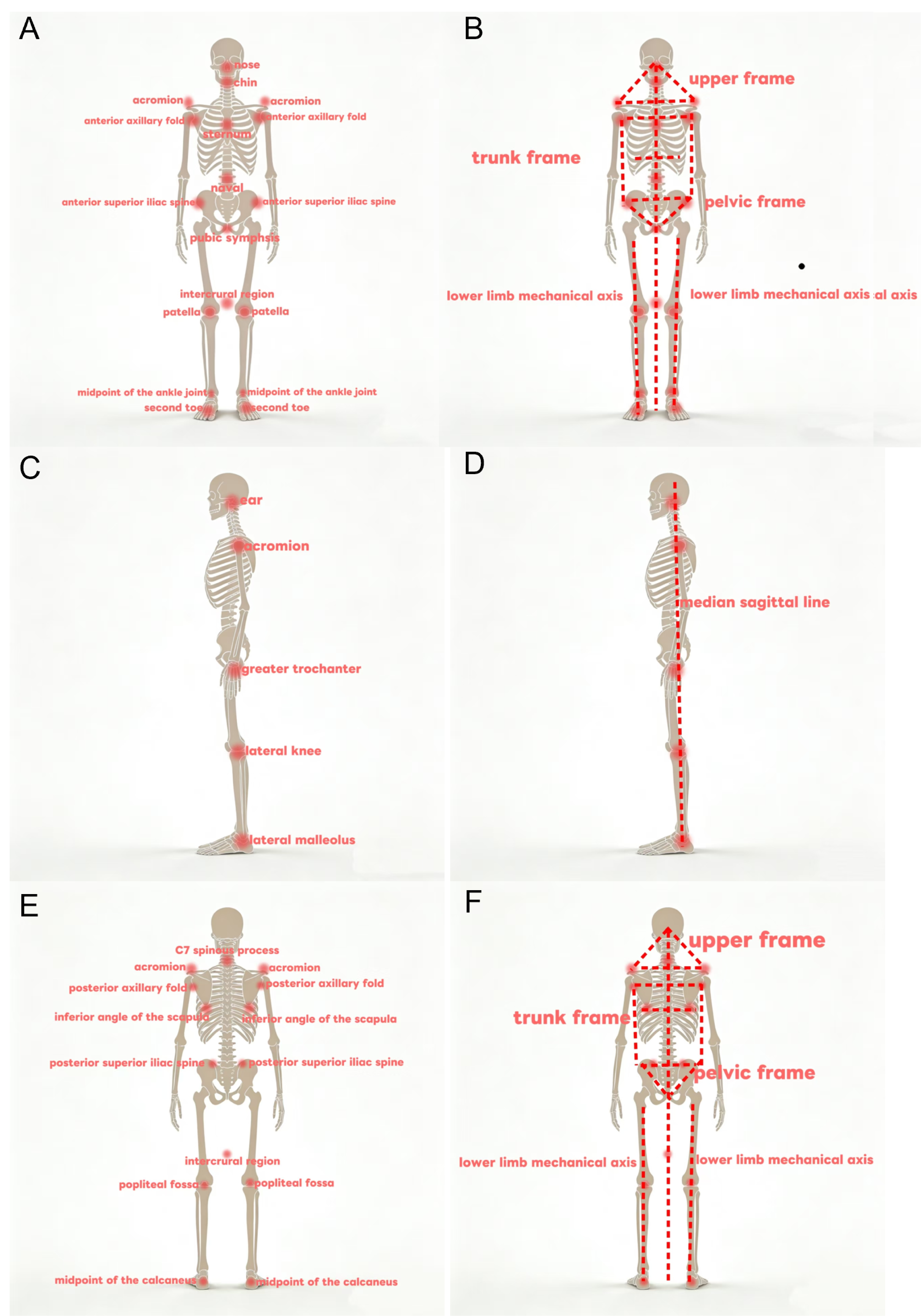

Supplement: Supplementary Figure 1 — Schematic illustration of surface marker placement and frame construction for three-dimensional trunk assessment. (A) Anterior view showing the placement of key surface markers. (B) Anterior view with markers connected to form the anterior reference frame, which allows clear visualization of coronal-plane deviations, including lateral translation of the pelvis, thorax, and head; within the frame concept, rotational abnormalities in the horizontal plane can also be inferred from the coronal-view image. (C) Lateral view showing the placement of key surface markers. (D) Lateral view with markers connected to form the sagittal reference frame, enabling assessment of sagittal-plane postural abnormalities, such as forward head posture, anterior pelvic tilt, and knee hyperextension. (E) Posterior view showing the placement of key surface markers. (F) Posterior view with markers connected to form the posterior reference frame, facilitating evaluation of posterior trunk alignment and asymmetry. [file Image1.tif]

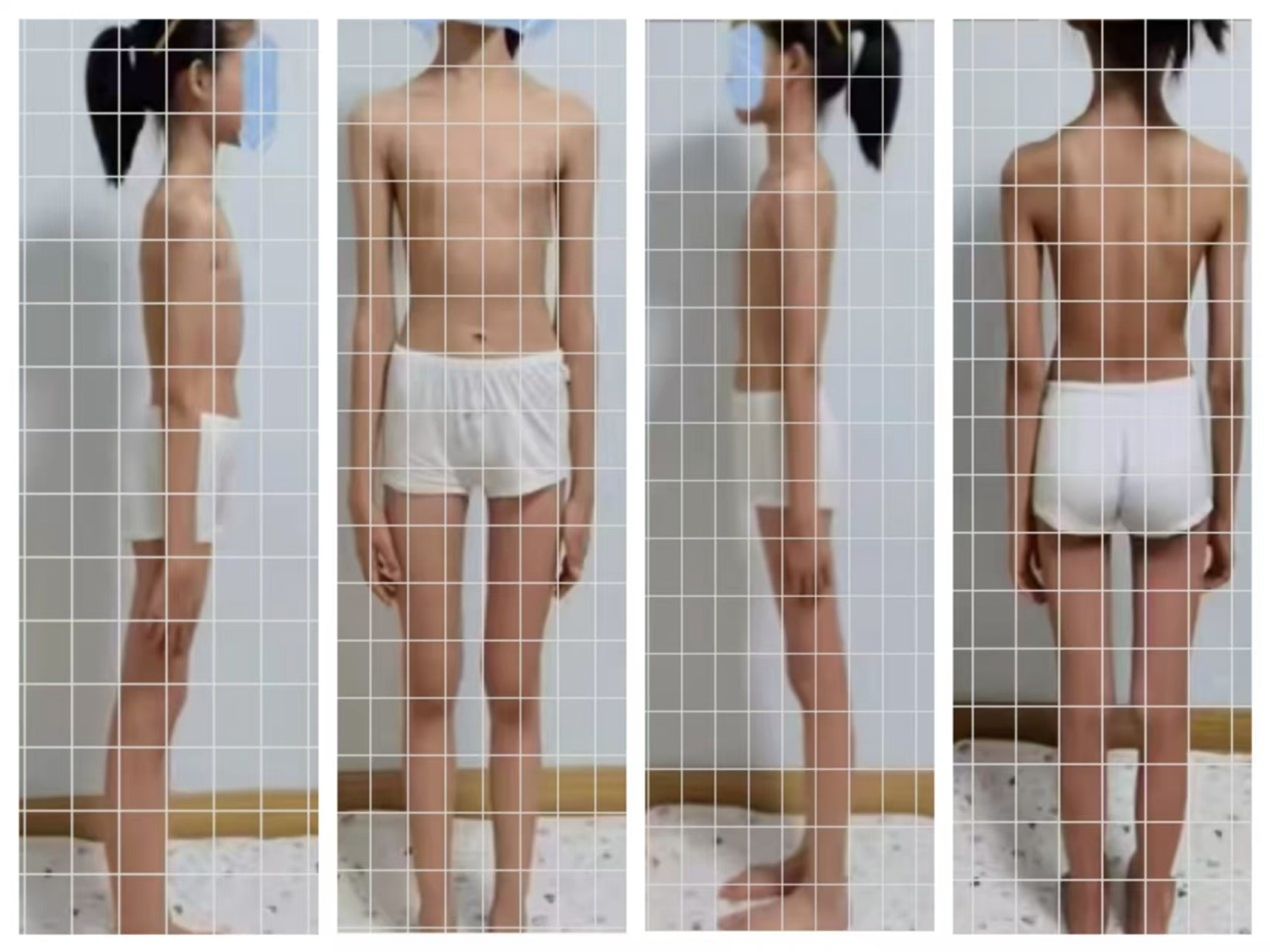

Supplement: Supplementary Figure 2 — Grid on photographs of coronal plane, horizontal plane and sagittal plane. [file Image2.tif]
